# Automatic Segmentation of Metastatic Breast Cancer Lesions on ^18^F-FDG PET/CT Longitudinal Acquisitions for Treatment Response Assessment

**DOI:** 10.3390/cancers14010101

**Published:** 2021-12-26

**Authors:** Noémie Moreau, Caroline Rousseau, Constance Fourcade, Gianmarco Santini, Aislinn Brennan, Ludovic Ferrer, Marie Lacombe, Camille Guillerminet, Mathilde Colombié, Pascal Jézéquel, Mario Campone, Nicolas Normand, Mathieu Rubeaux

**Affiliations:** 1 LS2N, University of Nantes, CNRS, 44000 Nantes, France; constance.fourcade@keosys.com (C.F.); Nicolas.Normand@univ-nantes.fr (N.N.); 2 Keosys Medical Imaging, 13 Imp. Serge Reggiani, 44815 Saint-Herblain, France; gianmarco.santini@keosys.com (G.S.); aislinn.brennan@keosys.com (A.B.); mathieu.rubeaux@keosys.com (M.R.); 3 CRCINA, University of Nantes, INSERM UMR1232, CNRS-ERL6001, 44000 Nantes, France; Caroline.Rousseau@ico.unicancer.fr (C.R.); Pascal.Jezequel@ico.unicancer.fr (P.J.); 4 ICO Cancer Center, 49000 Angers, France; Ludovic.Ferrer@ico.unicancer.fr (L.F.); Marie.Lacombe@ico.unicancer.fr (M.L.); camille.guillerminet@ico.unicancer.fr (C.G.); mathilde.colombie@ico.unicancer.fr (M.C.); mario.campone@ico.unicancer.fr (M.C.); 5 CRCINA, University of Angers, INSERM UMR1232, CNRS-ERL6001, 49000 Angers, France

**Keywords:** deep learning, automatic segmentation, metastatic breast cancer, imaging biomarkers, disease monitoring

## Abstract

**Simple Summary:**

In the recent years, several deep learning methods for medical image segmentation have been developed for different purposes such as diagnosis, radiotherapy planning or to correlate images findings with other clinical data. However, few studies focus on longitudinal images and response assessment. To the best of our knowledge, this is the first study to date evaluating the use of automatic segmentation to obtain imaging biomarkers that can be used to assess treatment response in patients with metastatic breast cancer. Moreover, the statistical analysis of the different biomarkers shows that automatic segmentation can be successfully used for their computation, reaching similar performances compared to manual segmentation. Analysis also demonstrated the potential of the different biomarkers including novel/original ones to determine treatment response.

**Abstract:**

Metastatic breast cancer patients receive lifelong medication and are regularly monitored for disease progression. The aim of this work was to (1) propose networks to segment breast cancer metastatic lesions on longitudinal whole-body PET/CT and (2) extract imaging biomarkers from the segmentations and evaluate their potential to determine treatment response. Baseline and follow-up PET/CT images of 60 patients from the EPICURE${}_{seinmeta}$ study were used to train two deep-learning models to segment breast cancer metastatic lesions: One for baseline images and one for follow-up images. From the automatic segmentations, four imaging biomarkers were computed and evaluated: SUL${}_{peak}$, Total Lesion Glycolysis (TLG), PET Bone Index (PBI) and PET Liver Index (PLI). The first network obtained a mean Dice score of 0.66 on baseline acquisitions. The second network obtained a mean Dice score of 0.58 on follow-up acquisitions. SUL${}_{peak}$, with a 32% decrease between baseline and follow-up, was the biomarker best able to assess patients’ response (sensitivity 87%, specificity 87%), followed by TLG (43% decrease, sensitivity 73%, specificity 81%) and PBI (8% decrease, sensitivity 69%, specificity 69%). Our networks constitute promising tools for the automatic segmentation of lesions in patients with metastatic breast cancer allowing treatment response assessment with several biomarkers.

## 1. Introduction

Breast cancer is the most common cancer in women worldwide, and approximately 34% of these women develop metastases [1]. As of today, patients with metastatic breast cancer have a median survival time of between 12 and 30 months [2]. They endure life-long treatments and are regularly monitored for disease progression.

Over the years, several standardized imaging-based criteria have been developed to assess treatment response in oncology. Response Evaluation Criteria in Solid Tumors (RECIST 1.1) with measurements on contrast-enhanced computed tomography (CT) and/or magnetic resonance imaging (MRI) [3] is the most widely used criteria in clinical practice and in clinical trials [4]. However, CT/MRI imaging alone does not have a good sensitivity to assess bone lesions [5]. As bone is the most common site of metastasis for breast cancer, alternative criteria and imaging modalities are considered for patients with metastatic breast cancer. For example, 18F-FDG positron emission tomography combined with computed tomography (PET/CT) evaluated according to PET Response Evaluation Criteria in Solid Tumors (PERCIST) [6] shows better prediction of overall survival (OS) and progression-free survival (PFS) compared to RECIST 1.1. [7]. However PERCIST, as with RECIST 1.1, only evaluates quantitatively a limited number of target lesions that are representative of tumor burden. To obtain quantitative data on all lesions, a segmentation of all metastases can be useful to extract for example information on imaging biomarkers such as (1) tumor metabolic activity with SUL${}_{peak}$ or SUV${}_{mean}$, (2) tumor volume with Metabolic Tumor Volume (MTV) or, (3) both with Total Lesion Glycolysis (TLG). These imaging biomarkers have been identified as promising prognostic factors for many diseases [8,9] and can be used to assess treatment response [10]. Unfortunately, manual segmentation is time consuming and too tedious to be performed in clinical practice, particularly when patients present many metastases [11]. This motivates the development of automatic methods for tumor segmentation.

Over the years, several automatic and semi-automatic methods based on computer vision were developed to segment lesions on PET images, but their performances are severely affected by low intensity contrast and tumor heterogeneity [11]. Since 2015 and the emergence of deep learning techniques for medical imaging, algorithms used to segment lesions on different modalities and/or for several diseases or anatomic regions started to outperform conventional methods [12,13]. PET/CT multi-modality fully convolutional neural networks (CNN) were proposed for different segmentation tasks including for lung cancer [14], bone lesions [15,16] and head and neck tumors [17,18]. However, most of these deep-learning techniques focus on a single acquisition, while lesion segmentation on multiple time points is required to assess treatment response. Recently, methods were developed for the monitoring of multiple sclerosis lesions [19,20,21] and the assessment of rectal cancer response [22] on longitudinal MRI images. Denner et al. [19] proposed a U-Net with input channels for each acquisition and an auxiliary self-supervised registration task to guide lesion segmentation. Jin et al. [22] used two networks (one for each acquisition) linked by a sub-network for response prediction. These techniques are however only applied to MRI and for a specific anatomical region, while our goal is to segment metastatic lesions on whole-body PET/CT.

The aim of this study was therefore to find a solution to automatically detect and segment breast cancer metastatic lesions on longitudinal whole-body PET/CT and obtain segmentations that can be used to compute imaging biomarkers for response assessment. To this end, we proposed two networks: (1) a U-Net for the segmentation of baseline acquisitions with two input channels for PET and CT and, (2) a U-Net for the segmentation of follow-up acquisitions with four input channels, two for the follow-up PET/CTs and two for the baseline PET and baseline lesion segmentation. Then we analyzed four imaging biomarkers to explore their potential for treatment response assessment.

Our main contributions are:

1. Development of a deep learning network to segment breast cancer metastatic lesions on baseline acquisitions with whole-body PET/CT images as input. Our network achieved a mean dice score of 0.66.
2. Development of a deep learning network to segment breast cancer metastatic lesions on follow-up acquisitions with whole-body PET/CT images as input. The difference of this network compared to the previous one lies in the use of baseline PET images and lesion segmentations as complementary inputs to the follow-up PET/CT images. This allows a better segmentation of the follow-up lesions that often present a lower contrast due to treatment response. Our network achieved a mean dice score of 0.58.
3. Automatic computation of 4 biomarkers from the automatic segmentation: (1) SUL${}_{peak}$ to assess metabolic changes, (2) TLG to determine metabolic and volume changes, (3) PET Bone Index (PBI) and (4) PET Liver Index (PLI), which estimates the lesion volume of the two sites most affected by metastatic breast cancer (bone and liver) [23]. We obtained good Lin’s concordance correlation coefficients (≥0.90) and Spearman’s rank correlation coefficients (≥0.80) between biomarkers computed on automatic segmentation and on manual segmentation.
4. Automatic assessment of patients’ treatment response using the previously defined biomarkers computed on the different PET/CT acquisitions. The SUL${}_{peak}$, with a 32% decrease between baseline and follow-up, was the biomarker best able to assess patients’ response (sensitivity 87%, specificity 87%).

## 2. Materials and Methods

### 2.1. Dataset

This work used the baseline and follow-up PET/CT images of 60 patients included in the prospective EPICURE${}_{seinmeta}$ metastatic breast cancer study (NCT03958136). The EPICURE${}_{seinmeta}$ study was approved by the ANSM (2018-A00959-46) and the CPP IDF I, Paris, France (CPPIDF1-2018-ND40-cat.1). A written informed consent was signed by each participant [24].

Images were acquired at two sites (A-ICO, N-ICO). At the A-ICO site, imaging was acquired with a Philips Vereos or a GE Discovery PET/CT imaging system; at the N-ICO site, with two different dual-slice Siemens Biograph PET/CT. Each patient had one baseline and one or two follow-up acquisitions: 60 baseline and 104 follow-up PET/CT images were available for this study. Baseline PET/CTs were acquired before initiation of a new treatment. Median time interval was 1.6 months (range: 0.9–7.4) between the baseline and the first follow-up image and 2.8 months (range: 1.8–11.1) between the baseline and the second follow-up.

Manual segmentation of all lesions was performed by one expert at A-ICO and by two experts (one for the baseline and one for the follow-ups) at N-ICO. Over 2000 lesions were segmented on baseline acquisitions. Two experts (one from each site) assessed treatment response at each follow-up time point according to the PERCIST criteria [6].

To evaluate the performance of our networks on unseen data, 10 patients (five from each site) with one baseline and one follow-up acquisition were also included.

### 2.2. Metastatic Lesion Segmentation

**Figure 1:**
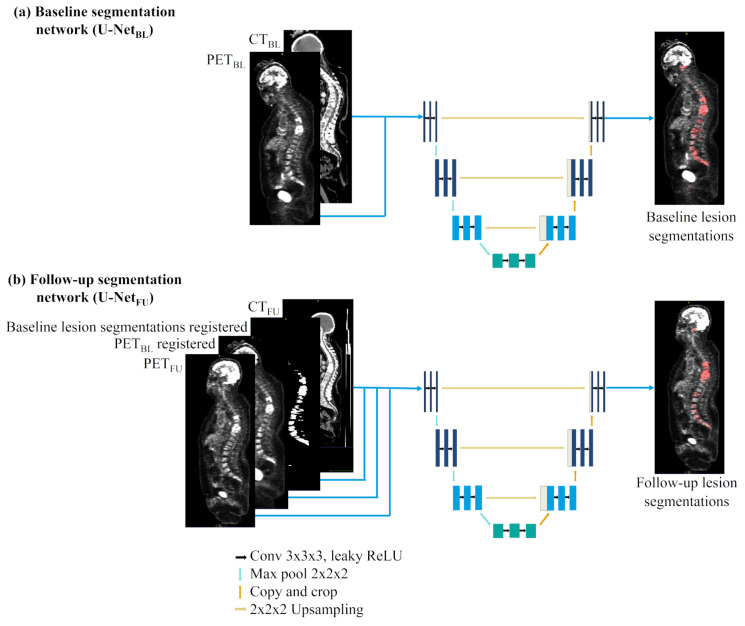
(**a**) U-Net${}_{BL}$ and (**b**) U-Net${}_{FU}$ networks’ architectures and inputs.

We proposed two networks based on the recently published 3D U-Net implementation called ”no new U-Net” (nnU-Net) (Implementation freely available on github: https://github.com/MIC-DKFZ/nnUNet, 15 December 2021) [25]: one network for the segmentation of baseline images called U-Net${}_{BL}$ and another one for the segmentation of follow-up acquisitions called U-Net${}_{FU}$. Figure 1 shows the architecture of the two networks and their input images. The nnU-Net achieved state of the art performance in several segmentation challenges such as KiTS2019 and the Medical Segmentation Decathlon [26,27]. It allows the automatic configuration of several hyper-parameters depending on given information such as data feature input or memory consumption requirements. The loss was defined as:

$$L_{Total}=L_{Dice}+L_{CE}$$

$L_{Dice}$ is the multi-class Dice loss as in [28] and $L_{CE}$ the cross entropy loss as in [29]. We used a stochastic gradient descent with an Adam optimizer and an initial learning rate of $3\times 10^{-4}$. Training ended after 1000 epochs (one epoch is defined as 250 batches). Elastic deformations, random scaling, random rotation, gamma augmentation were used as data augmentation during training. A more detailed list of the configurable parameters can be found in the original nnU-Net publication [25].

Deep learning experiments were performed using a NVIDIA GTX 1080 with 11 GB of RAM, with python 3.6 and pytorch 1.2.0.

Both networks were trained and validated using 5-fold cross-validation, with data balanced among folds in terms of acquisition site and number of follow-up images. For each network, we had 5 models trained and validated with different parts of the dataset. The U-Net${}_{BL}$ network was trained on baseline acquisitions and validated on baseline and follow-up acquisitions in order to obtain results that the ones from the U-Net${}_{FU}$ network could be compared to. For results to be comparable, the same patients’ folds were used to train and validate both networks. Validation with the U-Net${}_{BL}$ network on a specific patient’s follow-up acquisition was done with the model that was not trained with the baseline of this patient during cross-validation to avoid any bias.

CT images were resampled to match the size of PET image. PET images were converted to SUV${}_{BW}$ [30] to normalize lesion activity to the injected activity and body weight. SUV values were then clipped between 0 and 5 as a SUV ≥ 2.5 is a commonly used threshold indicative of malignancy [31] on 18F-FDG PET. All images were also resampled and normalized automatically during the nnU-Net preprocessing.

Baseline segmentation network (U-Net${}_{BL}$): With the U-Net${}_{BL}$ network only baseline images were used for training. The trained network was then validated on the 60 baseline images and 104 follow-up images according to the cross-validation scheme. The network had two inputs channels for PET and CT images.

Follow-up segmentation network (U-Net${}_{FU}$): The U-Net${}_{FU}$ network was trained and validated only with the 104 follow-up images, as baseline acquisitions were used as complementary input (see Figure 1b). When performing manual segmentation or assessing treatment response on follow-up images, experts usually look at both baseline and follow-up acquisitions to determine patients’ response. Indeed lesions are generally more visible on the baseline acquisition than on the follow-ups’ due to treatment response. Therefore, to mimic human behavior, two new input channels were added: one for baseline PET images and one for the lesion segmentations done manually on the baseline PET. To this end, baseline and follow-up PETs were rigidly registered using the ANTs pipeline with recommended settings [32]. The registration transformation was then applied to the baseline lesion segmentations. The network had four inputs channels for PET, CT, baseline PET and baseline lesion segmentation.

### 2.3. Segmentation Evaluation

To evaluate the results of each network, segmentation and lesion detection metrics were computed. For each network, validation performances were computed for its 5 models from the cross-validation training and then averaged. To test the performances of our networks on unseen data from both sites, models from the cross-validation training were combined in one single ensemble model.

Segmentation metrics: Two metrics based on the Dice score were used: the mean Dice score per acquisition and a global Dice score on all acquisitions combined as one. The Dice score evaluates the degree of overlap between the ground truth and the prediction. The mean Dice score is more affected by predictions errors when there are few lesions, while the global Dice score is more affected by errors when there are large lesions. For follow-up images, the difference in mean Dice score between the two networks was tested with a Wilcoxon signed-rank test (statistical significance = 0.001) as the Dice score distribution was not normal according to the Kolmogorov–Smirnov test (*p*-value ≤ 0.001).

Detection metrics: First the ground truth segmentation was separated in connected components to extract distinct lesions. Then, each ground truth lesion was overlapped with the global automatic segmentation: the lesion was considered detected (True Positive, TP) if the overlap was greater or equal to 50%, otherwise the lesion was counted as False Negative (FN). The same process was applied on the automatic segmentation: if the overlap between a lesion from the automatic segmentation and the global ground truth was less than 50%, the lesion was considered a False Positive (FP) [16]. This allows to compute the lesion detection recall ($\frac{TP}{TP+FN}$) and precision ($\frac{TP}{TP+FP}$).

### 2.4. Imaging Biomarkers

From the lesion segmentations obtained manually and automatically, four imaging biomarkers were computed: (1) SUL${}_{peak}$ to assess metabolic changes, (2) TLG to determine metabolic and volume changes, (3) PET Bone Index (PBI) and 4) PET Liver Index (PLI), which estimates the lesion volume of the two sites most affected by metastatic breast cancer (bone and liver) [23].

SUL${}_{peak}$: It is used to assess metabolic change between two acquisitions in the PERCIST criteria. PET images were converted to SUVLBM or SUL according to the Janmahasatian formulation [30]. A 1.2-cm–diameter spherical Volume of Interest (VOI) was centered on each voxel included in the segmentation. The mean SUL value of the sphere was then compared to previously included spheres, and the VOI with the highest mean SUL was kept. The SUL${}_{peak}$ was therefore not necessarily centered on the hottest voxel of the segmentation [33].

Total Lesion Glycolysis (TLG): It assesses both metabolic activity and lesion volume. After their automatic segmentation, lesions were tagged individually using a connected-component labeling. The TLG of each lesion was then calculated as:

$$TLG=MTV\times SUV_{mean}$$

*MTV* is the Metabolic Total Volume (lesion volume), and *SUV${}_{mean}$* (mean SUV value). The global *TLG* for each patient was computed as the sum of the TLGs of all their lesions.

PET Bone Index (PBI): In metastatic breast cancer, bone metastases are prevalent. They are associated with multiples painful complications like hypercalcemia, myelopathy, spinal cord compression or pathological fracture [23]. Different metrics were proposed to measure the extent of bone lesions. The Bone Scan Index (BSI) was found to be a response indicator for patients with castration-resistant metastatic prostate cancer [34] and can be used for patients with metastatic breast cancer [35]. This index is usually applied on Bone Scan with limited results for metastatic breast cancer [36], but can be successfully applied on PET [16]. After the automatic segmentation, bone lesions were labeled using a bone mask generated using a network presented in [37]. PBI was then calculated as the ratio of the bone lesion volume compared to the total bone volume.

PET Bone Index (PLI): In metastatic breast cancer, the liver is the second most frequently affected site and its metastases are known to be of poor prognosis [38]. PLI is similar to the PBI but for liver lesions. After their automatic segmentation, liver lesions were labeled using a liver mask generated using a CNN trained on the LiTS dataset [39]. PLI was then computed as the ratio of the liver lesion volume compared to the total liver volume.

Imaging biomarkers were measured on both manual and automatic segmentations. The normality of each imaging biomarker was tested using a Kolmogorov–Smirnov test (*p*-value ≤ 0.001), resulting in non-normal distribution for each. They were then compared with the Lin’s concordance correlation coefficient which evaluates the agreement between two variables [40], the Spearman’s rank correlation coefficient and a Wilcoxon signed-rank test (statistical significance = 0.001).

### 2.5. Response Assessment

To evaluate the potential of the biomarkers to assess treatment response, changes between the baseline and follow-up images were analyzed with each imaging biomarker (SUL${}_{peak}$, TLG, PBI and PLI). For this evaluation, baseline data was taken from the automatic segmentation done by the U-Net${}_{BL}$ network and follow-up data was taken from the segmentations done by the U-Net${}_{FU}$ network. The difference between baseline and follow-up images was measured as:

$$\Delta biomarker\left(\%\right)=\frac{\left(biomarkerFU-biomarkerBL\right)}{biomarkerBL}\times 100$$

with biomarkerBL, a biomarker taken on the baseline acquisition, and biomarkerFU, the same biomarker taken on the follow-up acquisition. To determine the best biomarker to assess treatment response, a Receiver Operating Characteristic (ROC) curve and its Area Under the Curve (AUC) were computed. PERCIST responses assessed by medical experts were binarized as responders for subjects with Complete Response (CR) and Partial Response (PR) and non-responders for subjects with Stable Disease (SD) or Progressive disease (PD). The ROC analysis evaluated each difference measured between baseline and follow-up acquisitions as a potential threshold for a binary prediction of treatment response. The ROC curve plots the True Positive Rate by False Positive Rate for all thresholds. The AUC represents the performance for the classification problems across all thresholds. The difference between two AUCs was tested using methods suggested by DeLong et al. [41], while the optimal cutoff value was determined using the Youden’s J statistic method [42]. For each imaging biomarker, we evaluated its correlation with treatment response by testing the statistical difference between responder and non-responder groups using a Mann-Whitney U test (statistical significance = 0.001).

## 3. Results

### 3.1. Metastatic Lesion Segmentation

**Figure 2:**
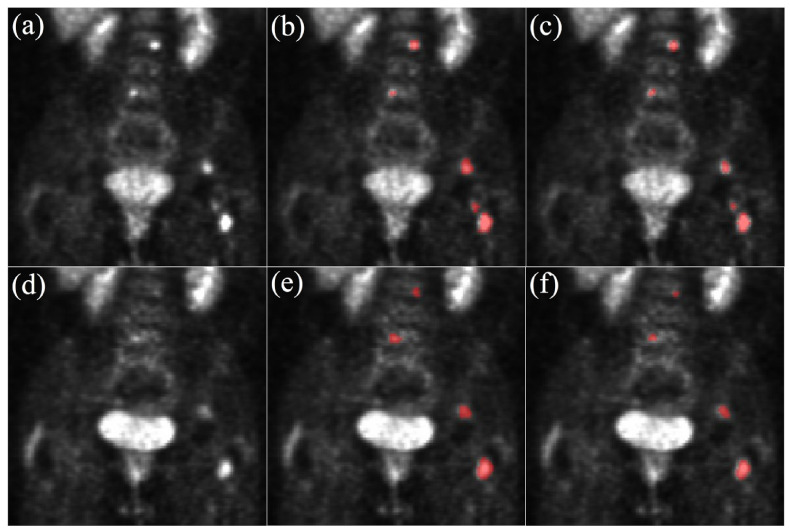
Segmentation examples on two acquisitions from the same patient. (**a**) PET BL, (**b**) GT BL, (**c**) U-Net${}_{BL}$, (**d**) PET FU, (**e**) GT FU, (**f**) U-Net${}_{FU}$. Zoom on the abdomen: kidneys, spine and bladder are visible. Due to the patient’s response to treatment, lesions on PET FU have a lower contrast than on PET BL and are less visible. BL = Baseline, GT = Ground Truth, FU = Follow-Up.

The U-Net${}_{BL}$ network was validated on baseline and follow-up acquisitions while the U-Net${}_{FU}$ network only validated only on follow-up acquisitions as baseline acquisitions were used as complementary inputs for this network. Segmentation evaluation was computed on the validation set of each patient’s fold to ensure a generalizable training. Overall segmentation results can be found in Table 1. An example of segmentation of lesions with a lower contrast in the follow-up acquisition compared to the baseline acquisition is presented in Figure 2. Both U-Net${}_{BL}$ and U-Net${}_{FU}$ networks had a mean Dice score between 0.50 and 0.66 and a global Dice score between 0.53 and 0.73. For the lesion detection task, the recall and precision were between 0.43–0.72 and 0.75–0.87 respectively. The confusion matrix for each network can be found in the Supplementary Materials (see Supplementary Table S1). On the follow-up acquisitions, the U-Net${}_{FU}$ network trained specifically for follow-up acquisitions with baseline images as input showed better performances (see Table 1) than U-Net${}_{BL}$ network trained only on baseline acquisitions. According to the Wilcoxon signed-rank test, the two mean Dice scores were statistically different (*p*-value ≤ 0.001).

**Figure 3:**
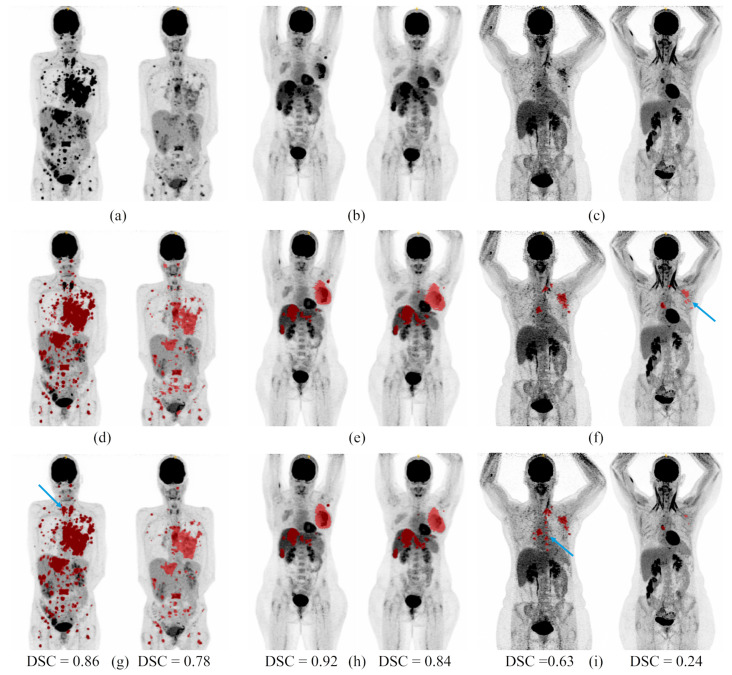
Segmentation examples on two acquisitions from 3 patients from the test dataset. (**a**–**c**): Maximum intensity projections of PET images. (**d**–**f**): Ground truth segmentation overlaid on the maximum intensity projections of PET images. (**g**–**i**): Automatic segmentation overlaid on the maximum intensity projections of PET images. U-Net${}_{BL}$ was used on the baseline acquisition and U-Net${}_{FU}$ on the follow-up acquisitions. For each pair of images: on the left the baseline acquisition and on the right the follow-up acquisition. DSC = dice score between the ground truth and the automatic segmentation. Blue arrows outline discrepancies between manual and automatic segmentations.

To evaluate our network on unseen data, we had accessed to 10 patients (five from each site) with one baseline and one follow-up acquisition. The results are presented in Table 1. Examples of lesion segmentations performed on both baseline and follow-up acquisitions for several patients from the test dataset are shown in Figure 3.

### 3.2. Imaging Biomarkers Measurements

**Figure 4:**
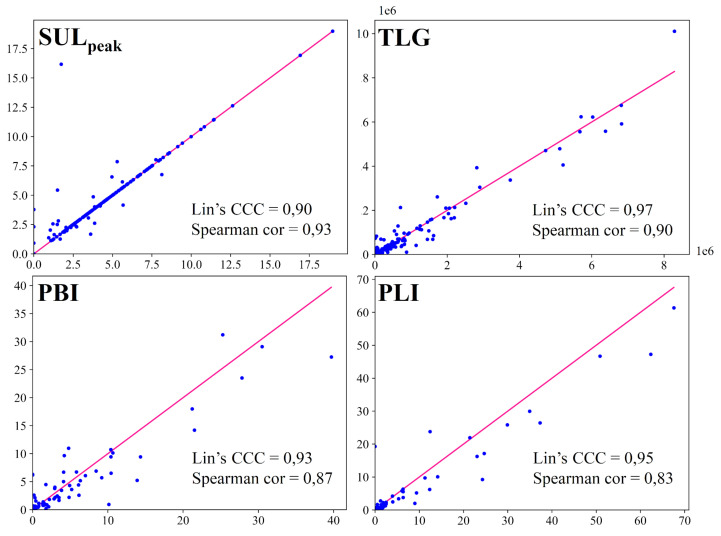
Graphical representation of each imaging biomarker with x axis biomarkers measured on ground truth segmentations and y axis biomarkers measured on automatic segmentations. The line represents perfect concordance. The concordance and the correlation are evaluated with the Lin’s concordance correlation coefficient (Lin’s CCC) and the Spearman’s rank correlation coefficient (Spearman cor) respectively.

The scatter plots for each imaging biomarker are shown in Figure 4, with the red lines representing the perfect concordance between automatic and manual biomarkers. The concordance correlation coefficients were 0.90, 0.97, 0.93, 0.95 and the Spearman’s rank correlation coefficients were 0.93, 0.90, 0.87, 0.83 for SUL${}_{peak}$, TLG, PBI and PLI respectively. The differences between manual and automatic biomarkers were not statistically significant with *p*-values equal to 0.06, 0.02, 0.01 and 0.01 for SUL${}_{peak}$, TLG, PBI and PLI respectively (statistical significance = 0.001).

### 3.3. Response Assessment

**Figure 5:**
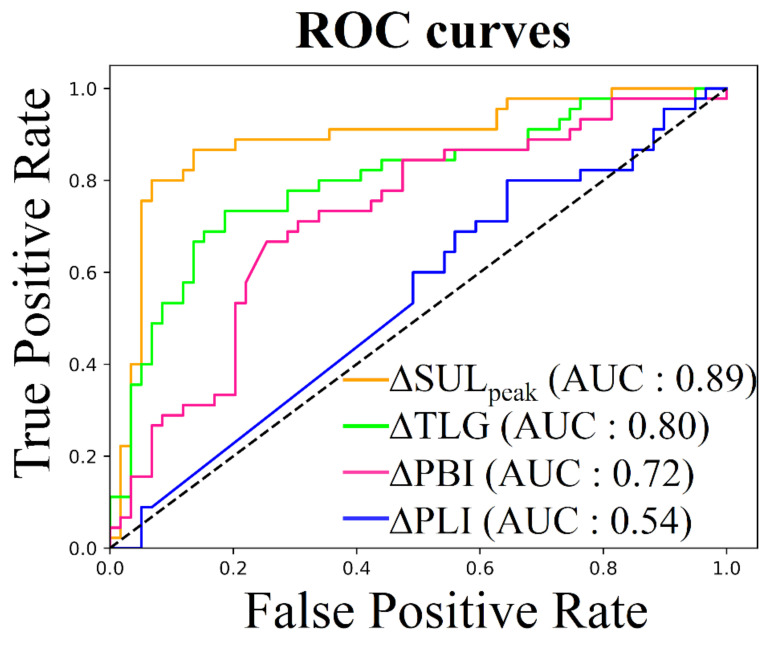
Receiver Operating Characteristic (ROC) curve, responders (CR or PR) vs. non-responders (SD or PD).

Figure 5 shows the ROC curve differentiating the responders from the non-responders for each imaging biomarker. The highest AUC score was obtained for ΔSUL${}_{peak}$ at 0.89, followed by ΔTLG at 0.80, ΔPBI at 0.72 and ΔPLI at 0.54 (Table 2). The AUCs for ΔSUL${}_{peak}$, ΔTLG and ΔPBI were not statistically different (*p*-values ≥ 0.001) but ΔPLI had significantly lower predictive value than the other biomarkers (*p*-value ≤ 0.001). The optimal cutoff values to classify patients as responders or non-responders were −32%, −43%, −8% and 0% for ΔSUL${}_{peak}$, ΔTLG, ΔPBI and ΔPLI respectively (Table 2). According to the Mann-Whitney U test, the responder/non-responder groups defined by each biomarker were statistically different (*p*-value ≤ 0.001) except for the ΔPLI (*p*-value = 0.062).

**Figure 6:**
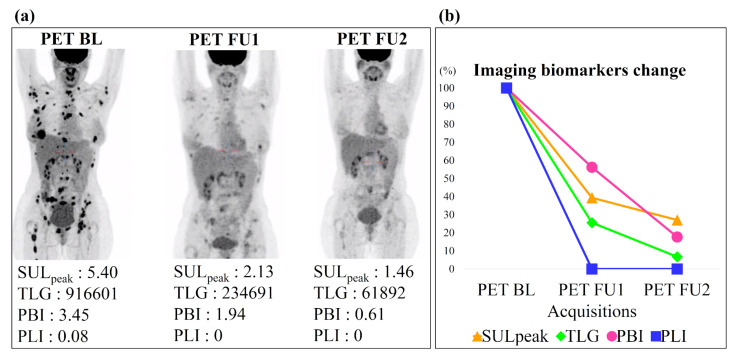
Imaging biomarkers assessment for one patient with partial response. (**a**) Maximum intensity projection of three PET acquisitions with their biomarkers measured using the automatic segmentation. (**b**) Graphical representation of each biomarker evaluation across 3 acquisitions (in percentage of the biomarkers from the baseline). BL for Baseline and FU for Follow-up.

Figure 6 shows an example of the imaging biomarkers automatically computed on three acquisitions of the same patient (one baseline and two follow-ups) and used to assess treatment response. This patient had a decrease at first and second follow-up (PET BL-PET FU1 and PET BL-PET FU2) of 61% and 73% for SUL${}_{peak}$, 74% and 93% for TLG, 44% and 82% for PBI, 100% and 100% for PLI. According to the cutoff values defined above, this patient was classified as responder by each biomarker, which is in agreement with the PERCIST evaluation performed by the expert.

## 4. Discussion

To the best of our knowledge, this is the first study to date evaluating the use of automatic segmentation to obtain imaging biomarkers that can be used to assess treatment response in patients with metastatic breast cancer. Indeed, most deep-learning studies on lesion segmentation aim to detect and segment lesions on a single acquisition for different purposes such as diagnosis, radiotherapy planning or to correlate image findings with other clinical data [43]. The statistical analysis of the different biomarkers shows that automatic segmentation can be successfully used for their computation, reaching similar performances compared to manual segmentation. Moreover, analysis demonstrated the potential of ΔSUL${}_{peak}$, ΔTLG and ΔPBI to determine treatment response.

Regarding the segmentation results on baseline images, our algorithm achieved performances comparable to previous studies on lesion segmentation with PET images. With only 60 patients and an important lesion heterogeneity in terms of location, size and contrast, we obtained a mean Dice score of 0.66 on the validation dataset. For comparison, Xu et al. [15] used a V-Net with two input channels to segment bone specific lesions on whole-body PET/CT from patients with multiple myeloma attained a mean Dice score of 0.69. In 2020, a challenge for automatic HEad and neCK TumOR (HECKTOR) segmentation in PET/CT was organized jointly with MICCAI, giving access to 201 patients for training and 53 for testing. Eighteen teams submitted their results to the challenge and the best method obtained a mean Dice score of 0.76 with a U-Net architecture with residual layers supplemented with squeeze and excitation normalization [17,44]. Another team tested the original 3D nnU-Net for this challenge and reached a Dice score of 0.72 [45]. Blanc-Durand et al. [46] also adopted a nnU-Net-based method to segment diffuse large B-cell lymphoma lesions on PET/CT from 733 patients and computed the total metabolic tumour volume. They obtained a mean Dice score of 0.73. Moreover, according to [47], in the context of head and neck tumor segmentation, the inter-observer Dice score in tumor segmentation only reach 0.69 on PET/CT, highlighting the challenge of lesion segmentation in one specific location. For metastatic lesions, the segmentation task is even more challenging for experts as tumor lesions can be of various sizes and located in different areas of the body.

Concerning the segmentation of follow-up images, our performances were less optimal with a mean Dice score of 0.58 on the validation dataset. However, results showed the usefulness of longitudinal segmentation as the U-Net${}_{FU}$ network with the baseline PET and baseline lesion segmentation as inputs had significantly better results than the U-Net${}_{BL}$ network (0.58 vs. 0.50). These results are in accordance with Denner et al. [19], who observed performance improvement with longitudinal networks using two time-points. In addition, on PET images, lesion contrast can be very different between two acquisitions, due to the patient’s treatment response, as shown in Figure 2. This makes the segmentation on follow-up images even more challenging and may explain the performance differences between baseline and follow-up acquisitions. Moreover, in follow-up images, many lesions are small, and under-segmented by the network due to lesions’ low contrast, which severely affects the Dice score [48]. This highlights the importance of using complementary metrics for evaluation, such as detection recall and precision to also show the performance of the network for lesion detection.

The evaluation of both networks with unseen data suggested that networks were not overfitted during training as we obtained dice scores between 0.63 and 0.77. However, limited data were used for testing as we had access to only 10 unseen patients with one baseline and one follow-up acquisition. Moreover, the test performances were computed using the ensemble model of each network, contrary to the validation performances computed with one model for each cross-validation fold and then averaged. This could explain the superior performances on the test dataset.

Overall, this work demonstrates that deep-learning-based segmentation can reach promising performances for metastatic breast cancer on longitudinal PET/CT. Furthermore, with this kind of segmentation imaging biomarkers can be computed and used to assess patients’ response to treatment. In a previous study, Choi et al. [10] investigated several imaging biomarkers, including ΔTLG computed on a manual segmentation of primary tumors from patients with stage II or III breast cancer, and reported an AUC of 0.76. In another study, Hatt et al. [49] reported an AUC of 0.79 and 0.91 for ΔSUL${}_{peak}$ and ΔTLG respectively. As for metastatic breast cancer, Goulon et al. [50] obtained AUCs of 0.96 and 0.82 for ΔSUL${}_{peak}$ and ΔTLG respectively. We observed similar AUCs with 0.89 for ΔSUL${}_{peak}$ and 0.80 for ΔTLG. Among the studied biomarkers, ΔSUL${}_{peak}$ showed a slightly better performance to predict response with a sensitivity of 87% and specificity of 87% for the optimal cutoff of −32%. This cutoff is close to the threshold used by the PERCIST criteria (−30%) to distinguish patients with partial response and stable disease. We chose to classify patients only as responders vs. non-responders, as we did not have access to enough patients in each PERCIST category. Yet, further analyses on a larger dataset could lead to the development of an automated computation of PERCIST response. Contrary to Hatt et al. [49], we did not find a statistical difference between ΔSUL${}_{peak}$ and ΔTLG. This may be explained by the fact that we used the PERCIST criteria to assess response, which depends on SUL${}_{peak}$, whereas they used histopathologic response.

ΔPBI and ΔPLI were never used to assess response to treatment or for prognostic purposes. Our study shows that ΔPBI has potential for treatment response assessment with an AUC of 0.72 not statistically different from ΔSUL${}_{peak}$ and ΔTLG, and a statistical difference between the responders and non-responders groups using the Mann-Whitney U test. This biomarker can however only be used for patients with bone metastases, and even though it is the most common metastatic location for patients with metastatic breast cancer, not every patient presents such metastases. Nonetheless, PBI, as it is inspired by BSI, could have some prognostic value. For example, Idota et al. [35] revealed that BSI may predict skeletal-related events in patients with metastatic breast cancer, so PBI may have the same potential. Our analysis of ΔPLI, on the contrary, did not reveal any power for response assessment with (1) an AUC of 0.54 statistically different from the three other biomarkers, and (2) no statistical difference between the responders and non-responders groups using the Mann-Whitney U test. However, liver metastases are known to be a poor prognostic factor and influence negatively Overall Survival (OS) and Progression Free Survival (PFS) [51]. Therefore, PLI may have some prognostic value for OS and PFS prediction.

This work has some limitations. Our study was performed on a relatively small dataset of 60 patients, and we did not have access to an external cohort for the validation of the networks. However, patients were recruited across two centers and over 2000 lesions were used to train each network, ensuring generalizable training. Results on the 10 unseen patients suggested that our networks were not overfitted during training. Moreover, all trainings were done using the same 5-fold cross-validation with data balanced among folds in terms of acquisition site and number of follow-up images. To avoid any bias and ensure valid comparison between networks, when validating the U-Net${}_{BL}$ network with follow-up acquisitions, we used the model that was not trained with the corresponding baseline acquisition. Since this prospective study is still ongoing, future included patients could be used to extend our test dataset and improve the validation of our networks. To overcome the lack of labelled data, pretraining with unlabeled data from our dataset but also from other PET/CT datasets could also be implemented as proposed by Alzubaidi et al. [52]. They were able to improve their results by about 10% in different classification scenarios and their work could be adapted to segmentation tasks. To do so, instead of assigning random labels as explored for classification, we could for instance try to pretrain the network to segment zones with SUV values superior to 2.5 (commonly used threshold indicative of malignancy).

## 5. Conclusions

The presented networks constitute promising tools for the automatic segmentation of malignant lesions in patients with metastatic breast cancer; segmentation from which information on imaging biomarkers can be extracted and used for treatment response assessment. Additional studies are needed to investigate the prognostic value of each imaging biomarker for OS and PFS prediction.

## Figures and Tables

**Table 1:** Quantitative evaluation for the two networks on baseline and follow-up acquisitions. (a) **Evaluation on validation data.** For each network, validation performances were computed for its 5 models from the cross-validation training and then averaged. (b) **Evaluation on test data.** For each network, models from the cross-validation training were combined in one single ensemble model and test performances were computed with this model. Only 10 unseen patients with one baseline and one follow-up acquisition were used.

Networks	Acquisitions	Mean Dice	Global Dice	Detection Recall	Detection Precision
U-Net${}_{BL}$	Baseline	0.66±0.19	0.73	0.72	0.87
	Follow-up	0.50±0.25	0.53	0.43	0.75
U-Net${}_{FU}$	Follow-up	0.58±0.24	0.64	0.63	0.78
**Networks**	**Acquisitions**	**Mean Dice**	**Global Dice**	**Detection Recall**	**Detection Precision**
U-Net${}_{BL}$	Baseline	0.78±0.17	0.84	0.67	0.92
	Follow-up	0.56±0.22	0.70	0.64	0.83
U-Net${}_{FU}$	Follow-up	0.66±0.15	0.77	0.75	0.88

**Table 2:** Biomarker for response assessment according to ROC analysis. Areas Under the Curve (AUCs) were computed on the ROC curve shown in Figure 5. The optimal cutoff value to differentiate between responder and non-responder patients was determined using the Youden’s J statistic method. Sensibility and specificity were computed for this optimal cutoff. P-values are determined using a Mann-Whitney U test for statistical difference between responder and non-responder groups defined by the optimal cutoff.

Biomarkers	AUC	Optimal Cutoff Value	Sensitivity	Specificity	*p*-Value
ΔSUL${}_{peak}$	0.89	−32%	87%	87%	≤0.001 *
ΔTLG	0.80	−43%	73%	81%	≤0.001 *
ΔPBI	0.72	−8%	69%	69%	≤0.001 *
ΔPLI	0.54	0%	53%	51%	≤0.001 *

* Statistically significant.

## Data Availability

The data are not publicly available as this is a private dataset.

## Supplementary Materials

The following are available online at https://www.mdpi.com/article/10.3390/cancers14010101/s1, Table S1: Confusion matrix for lesion detection.

## Abbreviations

The following abbreviations are used in this manuscript:

RECIST	Response Evaluation Criteria in Solid Tumors
CT	Computed Tomography
MRI	Magnetic Resonance Imaging
PET	Positron Emission Tomography
PERCIST	PET Response Evaluation Criteria in Solid Tumors
OS	Overall Survival
PFS	Progression-Free Survival
MTV	Metabolic Tumor Volume
TLG	Total Leson Glycolysis
CNN	Convolutional Neural Networks
TP	True Positive
FN	False Negative
FP	False Positif
PBI	PET Bone Index
PLI	PET Liver Index
VOI	Volume of Interest
ROC	Receiver Operating Characteristic
AUC	Area Under the Curve
CR	Complete Response
PR	Partial Response
SD	Stable Disease
PD	Progressive Disease
