# Supplementary material for: Automatic Segmentation of Metastatic Breast Cancer Lesions on 18F-FDG PET/CT Longitudinal Acquisitions for Treatment Response Assessment
Source: Cancers (Basel). 2021 Dec 26;14(1):101. doi: 10.3390/cancers14010101 (PMC8750371; doi:10.3390/cancers14010101)
Supplement: Supplementary file 1 [file cancers-14-00101-s001.zip › cancers-1480716-supplementary.pdf]

Table S1: Confusion matrix for lesion detection: (a) on validation data and (b) on test data. Ground truth segmentation was separated in connected components to extract distinct lesions. Each ground truth lesion was overlapped with the global automatic segmentation: the lesion was considered detected (True Pos-itive, TP) if the overlap was greater or equal to 50%, otherwise the lesion was counted as False Negative (FN). The same process was applied on the automatic segmentation: if the overlap between a lesion from the automatic segmentation and the global ground truth was less than 50%, the lesion was considered a False Positive (FP). False Negative lesions were not counted as they are not relevant for the task of lesions detection.

(a)

Methods	Acquisitions	TP Detection	FN Detection	FP Detection
U-Net$_{BL}$	Baseline	2026	769	312
	Follow-up	1337	1750	455
U-Net$_{FU}$	Follow-up	1872	1215	556

(b)

Methods	Acquisitions	TP Detection	FN Detection	FP Detection
U-Net$_{BL}$	Baseline	280	134	24
	Follow-up	211	120	43
U-Net$_{FU}$	Follow-up	249	82	34
